# Prospective associations between working alliance, basic psychological need satisfaction, and coaching outcome indicators: a two-wave survey study among 181 Dutch coaching clients

**DOI:** 10.1186/s40359-022-00980-9

**Published:** 2022-11-15

**Authors:** Margriet Vermeiden, Jennifer Reijnders, Eva van Duin, Marianne Simons, Mayke Janssens, Sanne Peeters, Nele Jacobs, Johan Lataster

**Affiliations:** 1 Faculty of Psychology, Department of Lifespan Psychology, Open Universiteit, P.O. Box 2960, 6401 DL Heerlen, The Netherlands; 2 Department of Psychiatry and Psychology, School for Mental Health and Neuroscience, Maastricht University Medical Centre, Maastricht, The Netherlands

**Keywords:** Working alliance, Self-determination theory, Basic psychological needs, Coaching, Counselling, Well-being, Longitudinal

## Abstract

**Background:**

The coach-coachee working alliance and coachee motivation seem important factors for achieving positive coaching results. Self-determination theory, specifically basic psychological need theory, has been proposed as a relevant framework for understanding these relationships. The current longitudinal survey study therefore investigates prospective associations between coachees’ appraisal of the working alliance, basic psychological need satisfaction, and the coaching outcome indicators goal attainment, wellbeing, absence of psychopathology, and personal growth initiative.

**Methods:**

The sample (N = 181) consisted of Dutch coachees that were recruited across a range of coaching settings and contexts. Online self-report questionnaires were administered twice (T_0_ and T_1_), with an intervening time of 3 weeks, assessing working alliance, basic psychological need satisfaction, goal attainment, wellbeing, absence of psychopathology, and personal growth initiative. Parallel analysis with Monte Carlo simulations and confirmatory factor analyses were performed to assess the dimensionality of working alliance and basic psychological need satisfaction scores. Multiple regression analyses (stepwise) were used to examine prospective (T_0_ to T_1_) associations between working alliance and basic psychological need satisfaction, and their association with outcome indicators.

**Results:**

The coachees’ perception of the working alliance was positively and reciprocally, although modestly, associated with basic psychological need satisfaction. In addition, both working alliance and basic psychological need satisfaction were prospectively associated with goal attainment, but not with other outcome indicators.

**Conclusions:**

Results provide tentative support for a role of basic psychological need satisfaction in facilitating the establishment of a good working alliance. Additionally, the perception of a good quality, need supportive relationship with the coach appears to be associated with better goal achievement, but not with other outcome indicators. Associations were generally modest, and more research is needed to better measure and comprehend the unique contributions of specific relational and motivational factors to outcomes in coaching and assess the robustness of the current study findings.

**Supplementary Information:**

The online version contains supplementary material available at 10.1186/s40359-022-00980-9.

## Background

Recent years have seen an exponential increase in the number of coaches worldwide [1, 2], and coaching has gained growing attention from industry and academia [3]. Coaching involves a diverse range of techniques and interventions [4], that take place in various settings (e.g., individual, team, organisation) [5] and contexts, including executive, health, and life coaching [6]. Therefore, coaching can hardly be captured in a comprehensive definition [7], although facilitating clients towards their goals [6, 8, 9], instead of leading them (as in psychotherapy), seems to be a universal factor that is present across coaching activities and conceptualizations [10]. Typically, this is a collaborative process oriented towards solutions and results [11], restricted to agreed and contracted goals, and for a predetermined period of time [6].

Williams [12] qualifies coaching as “[…] a multi-disciplinary, multi-theory synthesis and application of applied behavioural change” (p. 228). By assembling various effective components, coaching is establishing its own evidence base [12], which is important to enhance the conceptual understanding of coaching [7]. Because of the highly heterogeneous nature of coaching, research on its effective components has focused both on factors that are specific to certain coaching techniques or interventions, as well as on factors that are common across various forms of coaching [13–15]. Among the latter factors, the relationship between coach and client (hereafter: coachee), and the motivation of the coachee, have been repeatedly forwarded as key “common” factors for positive coaching outcomes [6, 15–17], sparking interest in the study of underlying psychological mechanisms. Several authors (e.g., [18–22]) have suggested that self-determination theory (SDT; [23])—a macro theory of human motivation, development, and health [23]—may be a particularly useful framework from which to understand the linkage between the coach-coachee relationship and coachee motivation, and how these factors relate to coaching outcomes. However, empirical studies are so far scarce, and the current study therefore aims to add to the empirical literature on the link between the coach-coachee relationship and coaching outcomes, viewed from a SDT perspective on coachee motivation.

### The working alliance in coaching

Although the coaching relationship is no clear-cut construct [24–27], research tends to converge on the importance of collaboration and consensus between coach and coachee, as originally recognized in Bordin's [28] conceptualization of the (therapeutic) “working alliance” [16, 24, 25]. In the coach-coachee working alliance, collaboration is an active process (i.e., both coach and coachee actively contribute) to negotiate and establish goals and set tasks to realise the goals mutually agreed upon [29], building on a bond of trust. This bond is a close connection between coach and coachee, consisting of feelings and attitudes needed to purposefully advance the process in order to achieve a positive coaching outcome [30]. The coach’s and coachee’s perception of the working alliance do not necessarily correspond [25, 31, 32], and the coachee’s appraisal of the working alliance possibly reflects, in part, the coachee’s ability of relating well to the coach [33].

The working alliance has been linked to positive coaching outcomes in various domains [26, 34, 35]. More specifically, recent meta-analytic findings [26] across 27 studies suggest consistent positive associations between working alliance quality and, most notably, affective, and cognitive coaching outcomes, as well as positive associations with results outcomes, and negative associations with unintended negative effects. In addition, findings from this meta-analysis [26] and previous studies (e.g., [36]) suggest that associations with outcomes tend to be strongest when the working alliance is rated from the coachee vs. from the coach perspective, in line with findings in psychotherapy settings [37, 38]. Coachee motivation may play an important role in explaining these results, as it has been forwarded as an important antecedent for establishing a working alliance [39], and because a strong working alliance may in turn fuel motivation for transferring knowledge outside the coaching sessions [40]. Thus, it seems relevant to zoom in on the interplay of relational and motivational factors in coaching. SDT has been forwarded as a useful framework to help understand these processes (e.g., [18–22]).

### A self-determination perspective on the working alliance

SDT is an extensively researched and applied theory [41] of human motivation [19, 21, 23, 41–48], is proven valid across cultures [21, 41, 43, 49, 50], and has practical value in many areas [41]. SDT has been applied in clinical and non-clinical settings (see: [18, 51] for qualitative studies in coaching) and is particularly useful for understanding human motivation in attaining goals [19, 21, 23, 42–48]. SDT is based on two premises: (i) the type of motivation rather than the amount of motivation is essential [23, 44], and (ii) a set of basic psychological needs (i.e., autonomy, competence, and relatedness) must be fulfilled for effective functioning. SDT distinguishes amotivation (i.e., absence of motivation), and different types of controlled and autonomous motivation, all leading to different outcomes [42, 45]. Autonomous motivation, in particular intrinsic motivation (i.e., performing an activity for its inherent contentment), is considered to affect the quality of behaviour, health, and wellbeing most positively, and is most likely to persist over time [44, 52].

According to SDT, satisfaction of the basic psychological needs—autonomy, competence, and relatedness—will encourage autonomous motivation [43]. The need for autonomy is considered most essential [49, 53] and refers to perceived volition in organising and regulating one’s own behaviour, giving a feeling of psychological freedom. The need for competence reflects a general tendency to be interested and determined in learning new skills to better adapt to challenges in different contexts. The need for relatedness represents a human predisposition toward social connection and security, and strongly impacts relationships [44, 54].

Several studies have suggested a central role for need support in effective coaching relationships [18–21, 51, 53], but the directionality of the association between basic psychological need satisfaction and working alliance quality in coaching has not been systematically investigated. According to relationships motivation theory (RMT; [55]), effective relationship-building is crucially driven by need satisfaction, but the reverse path may be equally probable, i.e., a well-established working alliance may play into the satisfaction of basic psychological needs (e.g., [19–21]), because it acknowledges the coachee as key agent in the change process (autonomy), considers his/her range of competencies and confidence to engage in certain activities (competence), and is based on an atmosphere of trust and genuine interest (relatedness) [21]. However, while theoretically plausible, and relevant for practitioners, the evidence base for the role of basic psychological need satisfaction as critical factor in the coaching relationship is currently scarce and based on cross-sectional investigations [18, 51, 53], prohibiting directional conclusions.

### The current study

The current longitudinal survey study aimed at furthering insight into the interplay of working alliance and basic psychological need satisfaction in coaching, and how these factors are prospectively associated with coaching outcomes. Because previous work in therapy and coaching settings has suggested that the client or coachee perspective is most crucially related to successful outcomes [17, 36, 37, 56] the focus in the current study is on the coachee’s appraisal of the working alliance, basic psychological need satisfaction and coaching outcome indicators. We included goal attainment as primary outcome due to the goal-oriented nature of coaching; presence of wellbeing and absence of psychopathology as separate indicators of overall mental health [57]; and personal growth initiative as an indicator of active and intentional involvement in personal change and development [58].

We expected positive, reciprocal associations between the coachees’ perception of the working alliance and basic psychological need satisfaction in coaching (hypothesis 1), and we expected the coachees’ perception of the working alliance and basic psychological need satisfaction to predict coaching outcome indicators, i.e., a higher working alliance quality and higher levels of basic psychological need satisfaction being predictive of more favourable outcomes (hypothesis 2).

## Methods

### Sample

The total sample consisted of 197 Dutch coachees who had agreed to participate in the study, with study entry criteria: (i) age ≥ 18 years, (ii) currently enrolled in or about to start a coaching trajectory, (iii) sufficient command of the Dutch language to understand instructions and provide informed consent. Demographic and other characteristics of the sample are presented in Table 1.

**Table 1:** Demographic and other characteristics of completers (N = 181)

Age, *M* (*SD*)	43.97 (11.2) range 20–77
*Gender, n (%)*	
Male	57 (31.5%)
Female	123 (68.0%)
Other	1 (0.6%)
*Education*^*a*^*, n (%)*	
Lower secondary	26 (14.4%)
Upper secondary	12 (06.6%)
Bachelor’s degree	96 (53.0%)
Master’s degree	41 (22.7%)
PhD	6 (3.3%)
*Work situation, n (%)*	
Full-time	64 (35.4%)
Part-time	83 (45.9%)
Unemployed or retired	23 (12.7%)
Other	11 (6.1%)
*Type of coaching, n (%)*	
Life coaching	84 (46.4%)
Career coaching	49 (27.1%)
Team coaching	9 (5.0%)
Health coaching	7 (3.9%)
Executive coaching	6 (3.3%)
Performance coaching	4 (2.2%)
Relationship coaching	4 (2.2%)
Family coaching	1 (0.6%)
Other	17 (9.4%)
*Contact with coach, n (%)*	
Mainly face to face	161 (89.0%)
Mainly online	9 (5.0%)
Mainly by telephone	2 (1.1%)
Other	9 (5.0%)
*Setting, n (%)*	
Individual	146 (80.7%)
Group	19 (10.5%)
Individual and group	16 (8.8%)
*Number of sessions completed at T0, n (%)*	
1–2 sessions	28 (15.5%)
3–5 sessions	72 (39.8%)
6–8 sessions	42 (23.2%)
9–11 sessions	14 (7.7%)
12–14 sessions	11 (6.1%)
≥ 15 sessions	14 (7.7%)
*Number of sessions between T0-T1, n (%)*	
0 sessions	50 (27.6%)
1 session	77 (42.5%)
2 sessions	33 (18.2%)
3–4 sessions	13 (7.2%)
5–6 sessions	4 (2.2%)
≥ 7 sessions	4 (2.2%)

^a^Highest educational level completed / note that percentages may not always add up exactly to 100.0% due to rounding

### Study design and procedure

The study followed a two-wave prospective design, requiring participants to fill out two nearly identical online self-report surveys with an interval of three weeks, during their coaching trajectory. Participants were recruited from July 2018 to December 2020 by psychology graduate students at Open Universiteit, via announcements on social media and other websites, and by asking coaching organisations and individual coaches to inform their coachees about the possibility to participate. After registration, participants received information about the objective, design, and procedure of the study, and were given the opportunity to have any questions answered. All participants were informed that participation was on a voluntary basis and gave digital informed consent at study entry. All aspects of the study, including the digital informed consent procedure, were assessed (against ethical and legal norms) and approved by the local Research Ethics Committee of Open Universiteit (#U2018/05422/HVM). The study was carried out in accordance with The Code of Ethics of the World Medical Association (Declaration of Helsinki) for medical research involving humans.

### Measures

Working alliance, basic psychological need satisfaction and the coaching outcome indicators goal attainment, wellbeing, (absence of) psychopathology, and personal growth initiative, were assessed at T_0_ (baseline) and T_1_ (follow-up). The T_0_ survey additionally covered demographic data and information about the coaching trajectory (see Table 1). The order of questionnaires was randomized across subjects to prevent order bias in the responses.

#### Working alliance

The short Working Alliance Inventory (hereafter: WAI-S) was used to assess the perceived quality of the working alliance between coach and coachee. The original 36 item working alliance inventory is widely used [24, 59–61] and has been proven reliable and valid in face-to-face and internet settings [62]. The current study used the validated Dutch 12-item version short form [63], derived from the original working alliance inventory [64], and adapted for coachees. The WAI-S measures the perceived agreement on goals (4 items), tasks (4 items), and perception of the affective bond (4 items), with previously reported scale reliabilities of, resp., Cronbach’s alpha = 0.83, 0.85, and 0.82 [63]. Examples of questions are: “My coach and I work together to determine the objectives for my coaching trajectory” (goals), “We agree on what is important for me to work on” (tasks), and “My coach and I respect one another” (bond). The extent to which each item reflects the thoughts or feelings of the coachee is assessed on a five-point Likert-type scale, ranging from *rarely or never* (1), *sometimes* (2), *often* (3), *very often* (4) to *always* (5). The current study used both the total scale, as global measure of working alliance quality, and the three subscales (hereafter: goals, tasks, bond).

#### Basic psychological need satisfaction

The Basic Psychological Needs in Coaching relationships questionnaire (hereafter: BPNs-COACH) was constructed to assess coachee basic psychological need satisfaction in the coaching context. The questionnaire combines a selection of items from the Basic Psychological Needs Scale (BPNS; [44, 65]), Basic Psychological Need Satisfaction and Frustration Scale (BPNSFS; [50]), and Health Care Climate Questionnaire (HCCQ; [66]). Coachees were asked to think about their coaching trajectory and indicate for a total of 15 statements (5 for each basic psychological need domain, i.e., autonomy, competence, relatedness), to what extent each statement reflected their experience, on a 6-point Likert-type scale, ranging from *never* (0), *rarely* (1), *sometimes* (2), *regularly* (3), *often* (4) to *always* (5). Items included “I was encouraged to make my own decisions” (autonomy), “I was strengthened in the belief in my own abilities” (competence), and “I felt understood” (relatedness). Preliminary data suggested a one-factor structure for the Dutch BPNs-COACH, with Cronbach’s α = 0.85 [67]. See Additional file 1, 2 for full questionnaire in Dutch and English. The current study used both the total scale, as global measure of basic psychological need satisfaction, and the three subscales (hereafter: autonomy, competence, relatedness).

#### Goal attainment

The primary outcome indicator, goal attainment, was assessed with a constructed four-item scale, based on previous studies to ensure validity: “The goal(s) of the coaching trajectory have been achieved (so far)” (based on [68]), “The coaching trajectory has (so far) changed me positively” (based on [63]), “The coaching trajectory has (had) a positive influence on my performance and development” (based on [17, 56]), and “How would you (so far) rate the overall coaching outcome?” (based on [17, 56]). Answers on the first three items were scored on a 7-point Likert-type scale, ranging from *totally disagree* (1) to *totally agree* (7). The last item was answered on a scale from 1 (*very poor*) to 10 (*very good*), rescaled to 1–7, in line with the other items. Previously [67], scores on the four items were combined in a scale score, with Cronbach’s α = 0.82.

#### Wellbeing

Wellbeing was measured with the Mental Health Continuum Short Form (MHC-SF; [57]), consisting of 14 items that assess emotional (3 items), psychological (6 items), and social wellbeing (5 items). Questions include “In the past month…” (or: “Since the previous assessment…”), “…how often did you feel interested in life?” (emotional wellbeing), “…how often did you feel that you liked most parts of your personality?” (psychological wellbeing), and “…how often did you feel that you belonged to a community (like a social group, your school, or your neighbourhood)?” (social wellbeing). Response options (6-point scale) range from *never* (0) to *everyday* (5), and the sum or weighted mean of item scores can be used as indicator of overall wellbeing [69]. Cronbach’s α for the total MHC-SF, which was used in the current study, was previously shown to be 0.89 in a Dutch population sample [70].

#### Absence of psychopathology

The Dutch 5 item Mental Health Inventory (MHI-5; [71]) was used to assess the absence of anxiety and depression symptoms [72]. The extent to which each question (e.g., “How much of the time, during the past month, have you been a very nervous person?”) applies, was rated on a 6-point scale ranging from *all of the time* (0) to *none of the time* (5)—higher scores thus indicating lower levels of psychopathology [73]. Following [72], item scores were summed and transformed linearly to a 0–100-point scale, a score of 100 indicating complete absence of psychopathology (see also: [72, 74, 75]). In a Dutch population sample, Cronbach’s alpha values for the total scale were shown to range from 0.78 to 0.81 [76].

#### Personal growth initiative

The Personal Growth Initiative Scale (PGIS; [58, 77]) assesses a person’s active involvement in change and development, expressed in nine statements, such as: “If I want to change something in my life, I initiate the transition process”. The amount of agreement with each statement is rated on a 6-point Likert-type scale ranging from *definitely disagree* (1) to *definitely agree* (6). Scores on the PGIS were shown to be unidimensional, with Cronbach’s α = 0.90 [58], and α = 0.88 for a previously used Dutch translation [67].

### Analysis

Data were analysed with R version 4.1.0 [78]. Negatively formulated items were reverse coded, scale scores were constructed, and Cronbach’s α values were calculated as indicators of scale reliability at T_0_ and T_1_. Demographic and other background variables (see Table 1), including possible differences between participants filling out both surveys (*completers*) and those who did at T_0_ but not at T_1_ (*dropouts*), were analysed. Main analyses were subsequently performed on data from completers only (N = 181).

For the adapted WAI-S and the newly constructed BPNs-COACH, following [79], parallel analyses with Monte Carlo simulations were conducted on the item scores at T_0_ to determine the number of factors to retain [80]. Appropriateness of factor analysis was determined using Bartlett’s test (significance value < 0.5), and sampling adequacy was assessed with a Kaiser–Meyer–Olkin (KMO) measure (values between 0.7 and 0.8 are considered good, and values between 0.8 and 0.9 are considered excellent [81, 82]). Factor loadings were examined, and rotation of factors with direct oblimin was applied when parallel analysis suggested to retain more than one factor. Confirmatory factor analyses (CFA) were subsequently performed on the item scores at T_1_ to verify the observed factor structure at T_0_ and compare it against theoretically expected factor structures (for BPNs-COACH: 3 latent factors representing autonomy, competence, and relatedness satisfaction [50]; for WAI-S: 3 latent factors representing goals, tasks, and bond [63]). Root mean squared error of approximation (RMSEA), comparative fit index (CFI), and standardized root mean squared residual (SRMR) were computed as indicators of model fit [83, 84].

Associations between working alliance and basic psychological need satisfaction at T_0_ and T_1_ were first explored using simple Pearson correlation coefficients. Next, to test hypothesis 1, multiple regression analyses were performed with basic psychological need satisfaction (T_1_) as dependent variable, predicted by working alliance (T_0_), and working alliance (T_1_) predicted by basic psychological need satisfaction (T_0_). To test hypothesis 2, multiple regression analyses were performed with, resp., goal attainment, wellbeing, absence of psychopathology, and personal growth initiative (T_1_) as dependent variables, predicted by working alliance (T_0_), and basic psychological need satisfaction (T_0_).

All regression analyses were performed stepwise, starting with a baseline model that included levels of the dependent variable at T_0_, age, gender (1 = male; 2 = female), educational level, number of coaching sessions completed at T_0_, and number of coaching sessions completed between T_0_ and T_1_ as independent variables. In a second step, basic psychological need satisfaction at T_0_ or working alliance at T_0_ were added separately as predictor variables. ANOVAs were performed to test whether adding predictors to the baseline model led to significant improvements in model fit. All findings were interpreted against a significance threshold of *p* < 0.05. We followed [85] for the interpretation of effect sizes, specifically: ΔR^2^ = 0.02 (small effect); ΔR^2^ = 0.13 (medium effect); ΔR^2^ = 0.26 (large effect). Where considered appropriate, false discovery rate or familywise error-corrected p-values (pFDR; pFWE, resp.) were reported alongside unadjusted p-values to account for multiple testing [86–88].

## Results

### Sample characteristics

Of the 197 participants that enrolled in the study, 181 completed both questionnaires (attrition = 8.1%). Table 1 presents demographic descriptives for this sample, in which women were overrepresented. Most participants had completed at least a bachelor’s degree, had a part-time or full-time job, and were enrolled in life coaching. Coaching sessions were mostly individual and face to face, and most participants had completed 3 to 5 sessions at T_0_ and 1 session between T_0_ and T_1_. Study dropouts were similar to completers on all characteristics except for being more likely of having a full-time job.

### Main study variables

Table 2 presents the mean scores, standard deviations, and Cronbach’s alpha values of the (sub)scales for working alliance, basic psychological need satisfaction and the outcome indicator variables, i.e., goal attainment, wellbeing, absence of psychopathology, personal growth initiative, at T_0_ and T_1_. Mean scores of total working alliance, basic psychological need satisfaction and their subscales were slightly higher, though not significantly, at T_1_ compared to T_0_. Mean scores for all outcome indicator variables were significantly higher at T_1_ compared to T_0_. Working alliance and basic psychological need satisfaction were significantly positively correlated, with Pearson Product-Moment correlation coefficients ranging from 0.55 to 0.78 at T_0_, and from 0.51 to 0.71 at T_1_, indicating (very) strong raw associations [85]; see Table 3.

**Table 2:** Main study variables at T_0_ and T_1_, for completers (N = 181)

	T_0_		T_1_		Test for difference between T_0_ and T_1_		
	*M (SD)*	α^*a*^	*M (SD)*	α^*a*^	*t-statistic (df)*	*p*^*b*^	*pFDR*
*Working alliance*							
Total	3.66 (0.65)	.93	3.71 (0.58)	.92	*t*(180) = − 1.72	0.088	.176
Goal	3.08 (0.63)	.80	3.15 (0.55)	.78	*t*(180) = − 1.90	0.059	.142
Task	3.12 (0.61)	.86	3.14 (0.56)	.85	*t*(180) = − 0.79	0.430	.469
Bond	3.32 (0.60)	.86	3.36 (0.58)	.86	*t*(180) = − 1.40	0.164	.245
*Basic Psychological Need Satisfaction*							
Total	3.78 (0.58)	.91	3.83 (0.55)	.92	*t*(180) = − 1.33	0.186	.247
Autonomy	3.26 (0.57)	.72	3.32 (0.53)	.73	*t*(180) = − 1.47	0.143	.245
Competence	3.16 (0.59)	.81	3.17 (0.60)	.85	*t*(180) = − 0.50	0.615	.615
Relatedness	3.56 (0.55)	.83	3.60 (0.51)	.85	*t*(180) = − 1.21	0.227	.273
*Outcome indicators*							
Goal attainment	4.49 (0.69)	.87	4.61 (0.61)	.88	*t*(180) = − 3.05	0.003	**.010**
Wellbeing	2.96 (0.73)	.89	3.12 (0.77)	.92	*t*(180) = − 4.12	< 0.001	** < 0.001**
Psychopathology (absence)	67.09 (14.77)	.83	69.02 (13.98)	.81	*t*(180) = − 4.90	< 0.001	** < 0.001**
Personal Growth Initiative	3.87 (0.65)	.85	4.01 (0.65)	.88	*t*(180) = − 2.82	0.005	**.016**

Values in bold represent significant findings when correcting for multiple testing

^a^Cronbach’s alpha; ^b^Two-tailed; pFDR = false discovery rate-corrected p-values, computed by ranking unadjusted p-values in ascending order, and multiplying each p-value by m/k, where k is the rank of the p-value, and m is the total number of tests

**Table 3:** Correlations between working alliance and basic psychological need satisfaction at T_0_ and T_1_, for completers (N = 181)

Variable	1	2	3	4	5	6	7	8	9	10	11	12
*Working alliance (T0)*												
1. Goal	–											
2. Task	0.83	–										
3. Bond	0.73	0.70	–									
*Working alliance (T1)*												
4. Goal	0.68	0.67	0.57	–								
5. Task	0.60	0.70	0.56	0.78	–							
6. Bond	0.58	0.57	0.76	0.64	0.63	–						
*Basic psychological need satisfaction (T0)*												
7. Autonomy	0.58	0.64	0.61	0.53	0.54	0.50	–					
8. Competence	0.56	0.62	0.55	0.54	0.56	0.47	0.72	–				
9. Relatedness	0.68	0.68	0.78	0.60	0.59	0.67	0.73	0.69	–			
*Basic psychological need satisfaction (T1)*												
10. Autonomy	0.49	0.53	0.50	0.62	0.63	0.56	0.59	0.54	0.58	–		
11. Competence	0.41	0.48	0.44	0.57	0.65	0.51	0.50	0.65	0.51	0.69	–	
12. Relatedness	0.53	0.56	0.61	0.64	0.70	0.71	0.53	0.55	0.69	0.75	0.70	–

All correlations *p* < *.001*

### Factor analysis and internal consistency estimates

#### WAI-S

Parallel analysis with Monte Carlo simulations indicated to retain one WAI-S-factor at T_0_, and factor analysis was considered appropriate; Bartlett’s test was significant (χ^2^ (66) = 1,573, *p* < 0.001) and KMO was 0.92. The single factor explained 57% of variance, and all items loaded satisfactory on this factor. CFA on item scores at T_1_ showed suboptimal fit for the one-factor model at T_1_, χ^2^ (54, *N* = 181) = 363.488, *p* < 0.001, RMSEA = 0.178, CFI = 0.766, SRMR = 0.090. The three-factor model (goals, tasks, bond) showed better fit, χ^2^ (51, *N* = 181) = 236.236, *p* < 0.001, RMSEA = 0.142, CFI = 0.860, SRMR = 0.078, Δχ^2^ (3) = 127.25 (p < 0.001) but was still suboptimal. Because the latent factors were highly correlated (0.708–0.964), and exploratory factor analysis (EFA) suggested retaining one factor, we used the global working alliance score in the regression models for testing hypothesis 1 and 2, for the sake of model parsimony. Whenever yielding significant results, further exploratory analyses were conducted with each of the subscales (goals, tasks, bond) as separate predictors.

#### BPNs-COACH

Parallel analysis with Monte Carlo simulations suggested to retain one BPNs-COACH-factor at T_0_, and factor analysis was considered appropriate; Bartlett’s test was significant (χ^2^ (105) = 1,370, *p* < 0.001) and KMO was 0.91. The single factor explained 44% of variance, and all but one item (“I felt insecure about my abilities”) loaded satisfactory on the single factor. CFA on item scores at T_1_ showed suboptimal fit for the one-factor model at T_1_, χ^2^ (77, *N* = 181) = 273.502, *p* < 0.001, RMSEA = 0.119, CFI = 0.851, SRMR = 0.066. The theoretically expected three-factor model (autonomy, competence, relatedness satisfaction) showed better fit, χ^2^ (74, *N* = 181) = 199.274, *p* < 0.001, RMSEA = 0.097, CFI = 0.905, SRMR = 0.064, Δχ^2^ (3) = 74.228 (p < 0.001). However, because the latent factors were again highly correlated (0.774–0.932), and EFA suggested retaining one factor, we used the global basic psychological need satisfaction score in the regression models for testing hypothesis 1 and 2. Whenever yielding significant results, further exploratory analyses were conducted with each of the subscales (autonomy, competence, relatedness satisfaction) as separate predictors.

### Multiple regression analyses

#### Hypothesis 1

Basic psychological need satisfaction at T_0_ significantly predicted working alliance at T_1_ (β(SE) = 0.23(0.07); 95% CI [0.09, 0.38]; *p* = 0.001; ΔR^2^ = 0.020), and vice versa, working alliance at T_0_ significantly predicted basic psychological need satisfaction at T_1_ (β(SE) = 0.17(0.08); 95% CI [< 0.01, 0.33]; *p* = 0.046; ΔR^2^ = 0.009). Separate exploratory regression analyses showed that working alliance at T_1_ was significantly associated with relatedness (β(SE) = 0.25(0.07); 95% CI [0.11, 0.40]; *p*_*FWE*_ = 0.002; ΔR^2^ = 0.024), and competence (β(SE) = 0.15(0.06); 95% CI [0.03, 0.26]; *p*_*FWE*_ = 0.048; ΔR^2^ = 0.011), but not with autonomy satisfaction at T_0_ (β(SE) = 0.12(0.06); 95% CI [< 0.01, 0.25]; *p*_*FWE*_ = n.s.; ΔR^2^ = 0.007), whereas none of the working alliance subscales at T_0_ were on their own significantly associated with basic psychological need satisfaction at T_1_ (goals: β(SE) = 0.09(0.07); 95% CI [ − 0.05, 0.24]; *p*_*FWE*_ = 0.567; ΔR^2^ = 0.002; tasks: β(SE) = 0.15(0.08); 95% CI [< − 0.01, 0.30]; *p*_*FWE*_ = 0.169; ΔR^2^ = 0.008; bond: β(SE) = 0.14(0.08); 95% CI [ − 0.02, 0.29]; *p*_*FWE*_ = 0.235; ΔR^2^ = 0.006).

#### Hypothesis 2

Working alliance at T_0_ significantly predicted goal attainment at T_1_, but was not associated with wellbeing, (absence of) psychopathology, or personal growth initiative at T_1_ (see Table 4). Separate exploratory regression analyses showed significant associations between T_1_ goal attainment and T_0_ working alliance bond (β(SE) = 0.20(0.07); 95% CI [0.08, 0.32]; *p*_*FWE*_ = 0.003; ΔR^2^ = 0.028) and tasks (β(SE) = 0.17(0.07); 95% CI [0.03, 0.31]; *p*_*FWE*_ = 0.047; ΔR^2^ = 0.014), but not with goals (β(SE) = 0.14(0.06); 95% CI [0.01, 0.26]; *p*_*FWE*_ = 0.091; ΔR^2^ = 0.011). Basic psychological need satisfaction at T_0_ significantly predicted goal attainment at T_1_, but was not associated with wellbeing, (absence of) psychopathology, or personal growth initiative at T_1_ (see Table 5). Autonomy (β(SE) = 0.19(0.06); 95% CI [0.07, 0.32]; *p*_*FWE*_ = 0.009; ΔR^2^ = 0.023), competence (β(SE) = 0.19(0.06); 95% CI [0.07, 0.32]; *p*_*FWE*_ = 0.008; ΔR^2^ = 0.023), and relatedness satisfaction at T_0_ (β(SE) = 0.16(0.07); 95% CI [0.04, 0.29]; *p*_*FWE*_ = 0.039; ΔR^2^ = 0.015) were all significant predictors of goal attainment at T_1_.

**Table 4:** Relationships between working alliance and coaching outcome indicators across T_0_-T_1_, adjusted for demographic factors, number of coaching sessions, and initial level of outcome variables

T_1_ Goal attainment	Step 1			Step 2			
	Adj. R^2^		*p*	ΔR^2^		*p*	*pFWE*
	.486		< .001	.023		< .001	** < .001**
	β(SE)	95% CI	*p*	β(SE)	95% CI	*p*	*pFWE*
Age	− .06 (.05)	[ − .17, .05]		− .03 (.05)	[ − .14, .07]		
Gender	− .08 (.11)	[ − .31, .15]		− .11 (.11)	[ − .33, .11]		
Education^a^	− .04 (.05)	[ − .15, .07]		− .05 (.05)	[ − .15, .06]		
№ sessions T_0_	.10 (.06)	[ − .02, .22]		.13 (.06)	[.01, .24]	.029	
№ sessions T_0_-T_1_	.11 (.05)	[< .01, .22]	.043	.10 (.05)	[< − .01, .21]		
T_0_ Goal attainment	.65 (.06)	[.53, .77]	< .001	.52 (.07)	[.38, .66]	< .001	** < .001**
T_0_ Working Alliance				.20 (.07)	[.07, .34]	.003	**.011**

T_1_ Wellbeing	Step 1			Step 2			
	Adj. R^2^		*p*	ΔR^2^		*p*	*pFWE*
	.582		< .001	− .002			
	β(SE)	95% CI	*p*	β(SE)	95% CI	*p*	*pFWE*
Age	− .08 (.05)	[ − .18, .01]		− .09 (.05)	[ − .18. .01]		
Gender	− .03 (.10)	[ − .23, .18]		− .02 (.10)	[ − .22, .19]		
Education^a^	− .14 (.05)	[ − .24, − .05]	.004	− .14 (.05)	[ − .24, − .04]	.006	**.022**
№ sessions T_0_	− .08 (.05)	[ − .18, .02]		− .08 (.05)	[ − .18, .02]		
№ sessions T_0_-T_1_	.08 (.05)	[ − .02, .18]		.08 (.05)	[ − .02, .18]		
T_0_ Wellbeing	.79 (.05)	[.69, .89]	< .001	.80 (.05)	[.70, .90]	< .001	** < .001**
T_0_ Working Alliance				− .03 (.05)	[ − .13, .07]		

T_1_ Psychopathology (absence)	Step 1			Step 2			
	Adj. R^2^		*p*	ΔR^2^		*p*	*pFWE*
	.870		< .001	< .001			
	β(SE)	95% CI	*p*	β(SE)	95% CI	*p*	*pFWE*
Age	− .02 (.03)	[ − .07, .04]		− .02 (.03)	[ − .07, .04]		
Gender	− .03 (.06)	[ − .14, .08]		− .03 (.06)	[ − .14, .09]		
Education^a^	.02 (.03)	[ − .04, .07]		.02 (.03)	[ − .04, .07]		
№ sessions T_0_	− .03 (.03)	[ − .09, .02]		− .03 (.03)	[ − .09, .02]		
№ sessions T_0_-T_1_	.01 (.03)	[ − .04, .06]		.01 (.03)	[ − .04, .06]		
T_0_ Psychopathology (absence)	.94 (.03)	[.88, .99]	< .001	.94 (.03)	[.88, .99]	< .001	** < .001**
T_0_ Working Alliance				− .01 (.03)	[ − .06, .05]		

T_1_ Personal growth initiative	Step 1			Step 2			
	Adj. R^2^		*p*	ΔR^2^		*p*	*pFWE*
	.205		< .001	− .001			
	β(SE)	95% CI	*p*	β(SE)	95% CI	*p*	*pFWE*
Age	− .03 (.07)	[ − .17, .10]		− .02 (.07)	[ − .16, .11]		
Gender	.05 (.14)	[ − .23, .32]		.03 (.14)	[ − .26, .31]		
Education^a^	− .06 (.07)	[ − .19, .08]		− .06 (.07)	[ − .20, .07]		
№ sessions T_0_	− .02 (.07)	[ − .15, .12]		.02 (.07)	[ − .16, .11]		
№ sessions T_0_ − T_1_	.18 (.07)	[.05, .32]	.007	.18 (.07)	[.05, .31]	.009	**.035**
T_0_ Personal growth initiative	.46 (.07)	[.33, .60]	< .001	.45 (.07)	[.31, .58]	< .001	** < .001**
T_0_ Working Alliance				.06 (.07)	[ − .08, .21]		

Values in bold represent significant findings when correcting for multiple testing

^a^Highest educational level completed; pFWE = family-wise error-corrected p-values, computed by multiplying the unadjusted p-value by the total number of outcomes (N = 4). ΔR^2^ is based on adjusted R^2^ values

**Table 5:** Relationships between basic psychological need satisfaction and coaching outcome indicators across T_0_-T_1_, adjusted for demographic factors, number of coaching sessions, and initial level of outcome variables

T_1_ Goal attainment	Step 1			Step 2			
	Adj. R^2^		*p*	ΔR^2^		*p*	*pFWE*
	.486		< .001	.029		< .001	**.003**
	β(SE)	95% CI	*p*	β(SE)	95% CI	*p*	*pFWE*
Age	− .06 (.05)	[ − .17, .05]		− .05 (.05)	[ − .15, .06]		
Gender	− .08 (.11)	[ − .31, .15]		− .10 (.11)	[ − .32, .12]		
Education^a^	− .04 (.05)	[ − .15, .07]		− .06 (.05)	[ − .17, .04]		
№ sessions T_0_	.10 (.06)	[ − .02, .22]		.12 (.06)	[< .01, .23]	.038	
№ sessions T_0_-T_1_	.11 (.05)	[< .01, .22]	.043	.10 (.05)	[< − .01, .21]		
T_0_ Goal attainment	.65 (.06)	[.53, .77]	< .001	.51 (.07)	[.36, .65]	< .001	** < .001**
T_0_ Basic Psychological Need Satisfaction				.23 (.07)	[.10, .36]	< .001	**.003**

T_1_ Wellbeing	Step 1			Step 2			
	Adj. R^2^		*p*	ΔR^2^		*p*	*pFWE*
	.582		< .001	− .002			
	β(SE)	95% CI	*p*	β(SE)	95% CI	*p*	*pFWE*
Age	− .08 (.05)	[ − .18, .01]		− .08 (.05)	[ − .18, .02]		
Gender	− .03 (.10)	[ − .23, .18]		− .03 (.10)	[ − .24, .17]		
Education^a^	− .14 (.05)	[ − .24, − .05]	.004	− .15 (.05)	[ − .25, − .05]	.004	**.015**
№ sessions T_0_	− .08 (.05)	[ − .18, .02]		.09 (.05)	[ − .18, .01]		
№ sessions T_0_-T_1_	.08 (.05)	[ − .02, .18]		.09 (.05)	[ − .02, .17]		
T_0_ Wellbeing	.79 (.05)	[.69, .89]	< .001	.78 (.05)	[.68, .89]	< .001	** < .001**
T_0_ Basic Psychological Need Satisfaction				.03 (.05)	[ − .08, .13]		

T_1_ Psychopathology (absence)	Step 1			Step 2			
	Adj. R^2^		*p*	ΔR^2^		*p*	*pFWE*
	.870		< .001	< .001			
	β(SE)	95% CI	*p*	β(SE)	95% CI	*p*	*pFWE*
Age	− .02 (.03)	[ − .07, .04]		− .02 (.03)	[ − .07, .04]		
Gender	− .03 (.06)	[ − .14, .08]		− .04 (.06)	[ − .15, .07]		
Education^a^	.02 (.03)	[ − .04, .07]		.01 (.03)	[ − .05, .06]		
№ sessions T_0_	− .03 (.03)	[ − .09, .02]		− .04 (.03)	[ − .09, .01]		
№ sessions T_0_-T_1_	.01 (.03)	[ − .04, .06]		.01 (.03)	[ − .05, .06]		
T_0_ Psychopathology (absence)	.94 (.03)	[.88, .99]	< .001	.93 (.03)	[.88, .99]	< .001	** < .001**
T_0_ Basic Psychological Need Satisfaction				.04 (.03)	[ − .01, .10]		

T_1_ Personal growth initiative	Step 1			Step 2			
	Adj. R^2^		*p*	ΔR^2^		*p*	*pFWE*
	.205		< .001	.003			
	β(SE)	95% CI	*p*	β(SE)	95% CI	*p*	*pFWE*
Age	− .03 (.07)	[ − .17, .10]		− .03 (.07)	[ − .16, .11]		
Gender	.05 (.14)	[ − .23, .32]		.02 (.14)	[ − .26, .30]		
Education^a^	− .06 (.07)	[ − .19, .08]		− .07 (.07)	[ − .20, .07]		
№ sessions T_0_	− .02 (.07)	[ − .15, .12]		− .03 (.07)	[ − .16, .11]		
№ sessions T_0_-T_1_	.18 (.07)	[.05, .32]	.007	.18 (.07)	[.04, .31]	.010	**.040**
T_0_ Personal growth initiative	.46 (.07)	[.33, .60]	< .001	.43 (.07)	[.29, .57]	< .001	** < .001**
T_0_ Basic Psychological Need Satisfaction				.09 (.07)	[ − .05, .24]		

Values in bold represent significant findings when correcting for multiple testing

^a^Highest educational level completed; pFWE = family-wise error-corrected p-values, computed by multiplying the unadjusted p-value by the total number of outcomes (N = 4). ΔR^2^ is based on adjusted R^2^ values

## Discussion

This study examined prospective associations between working alliance, basic psychological need satisfaction and coaching outcome indicators, as measured twice among Dutch coachees, during their coaching trajectory. Findings showed that coachees who reported a relatively higher (vs. lower) degree of basic psychological need satisfaction at baseline were significantly more likely to rate the working alliance with their coach more positively three weeks later. Vice versa, coachees who evaluated the overall working alliance at baseline more positively (vs. less positively) were also more likely to report a higher degree of basic psychological need satisfaction at follow-up, although this path was less pronounced. The data thus generally support our first hypothesis by suggesting that the coachees’ perception of the overall working alliance quality is positively and reciprocally, although modestly, associated with the experienced satisfaction of basic psychological needs in the coaching relationship.

When looking at how these two constructs were prospectively associated with coaching outcome indicators, we found that both working alliance quality and basic psychological need satisfaction at baseline were positively, although again modestly, associated with goal attainment at follow-up, but not with wellbeing, (absence of) psychopathology, or personal growth initiative. Our second hypothesis was therefore weakly supported, and only for the outcome domain of goal attainment.

### Reciprocal associations between working alliance and basic psychological need satisfaction

Although raw correlations between working alliance quality and basic psychological need satisfaction were relatively strong (r = 0.51 to r = 0.78), prospective reciprocal associations in the multiple regression models were quite modest. On top of the variance explained by levels of the dependent variable at baseline, demographic factors, and number of coaching sessions, basic psychological need satisfaction explained 2% of variance in working alliance quality at follow-up. The other way around, working alliance quality at baseline explained merely 0.9% of variance in basic psychological need satisfaction at follow-up. Although the confidence interval for this association did not include zero, its lower bound was < 0.01, with corresponding *p*-value = 0.046, thus not providing strong evidence against the null hypothesis [89, 90]. Indeed, none of the separate working alliance factors at baseline were significantly associated with basic psychological need satisfaction at follow-up. Thus, while our data point to a (weak) prospective path from basic psychological need satisfaction to working alliance quality during coaching trajectories, the evidence for a reverse path seems less substantial.

Exploratory analyses suggested that, among the three basic psychological needs at baseline, satisfaction of the need for relatedness was most predictive of perceived working alliance quality at follow-up. Feeling related to one’s coach in terms of rapport, trust, and support has been previously underlined as quintessential for a successful coach-coachee alliance [91]. The need for relatedness was measured by five items of BPNs-COACH: “I felt understood”; “I had the feeling that I had only superficial contact (with my coach)” (reverse coded); “I felt I was cared for”; “I was treated cold and distant” (reverse coded); “I was accepted for who I was/am”. These items reflect critical components of a good working relationship as formulated by the Rogerian theory [92], namely unconditional positive regard, empathy and, to a lesser extent, congruence. Recent reviews endorsed these components as most important [93–96] and effective [97] in building a sturdy basis for a coaching trajectory, and our findings seem to support this.

### Associations with outcome indicators

#### Working alliance and goal attainment

On top of the variance explained by a priori covariates, coachees’ rating of working alliance quality at baseline explained 2.3% of variance in goal attainment at follow-up. This association was significant yet rather modest, and not fully in line with previous meta-analytical findings [26], which suggest working alliance quality to be moderately—not weakly—associated with goal attainment (r = 0.32). However, these meta-analytical estimates have been based on aggregated product-moment correlations from mostly cross-sectional studies, thus not directly comparable to findings from the current prospective study. Indeed, more rigorous longitudinal analyses in a therapeutic context have shown similarly modest contributions of early session working alliance quality to outcome variance (1.8%–4.7%; [98]), in line with current results. Thus, while findings from different studies generally align in demonstrating a consistent relationship between working alliance quality and (results) outcomes, the strength of this relationship ranges from weak to moderate, likely depending on specific aspects of individual studies, such as the setting, the research design, and the method of data analysis (see also: [26]).

Among the working alliance factors, the perception of an affective bond with the coach was suggested to be most predictive of goal attainment. Previous work [34, 56] has instead suggested that the perceived agreement on goals and tasks is most important for coaching outcomes. This discrepancy in results may be explained by differences in study designs and analytical approaches, and types of coaching examined. Most notably, whereas other studies [34, 56] focused on executive and workplace/career coaching, our study purposely included various types of coaching, among which life coaching (46.4%) was overrepresented. While all these types of coaching aim to stimulate personal development, executive and workplace coaching may be more directly focused on performance or skills enhancement, whereas life coaching typically has a more holistic, explorative perspective on general life enhancement [99]. According to Bordin’s theory, various settings may demand and generate different types of alliance that may result in various patterns and scores on tasks, goals, and bond aspects [100, 101], and the bond factor may be more predictive of outcomes in some settings than others. Also, the time at which the working alliance is assessed (i.e., sooner or later in the trajectory) is a known factor to affect results [25, 102] and may partly explain discrepancies between study findings. While task and goal components tend to increase linearly over sessions, development of the bond component is typically more complex [103].

Another important point of attention are the mixed results regarding the dimensionality of the working alliance assessed with the working alliance index. Exploratory factor analysis of our data suggested one global working alliance factor, yet confirmatory factor analysis favoured a three-factor model that distinguished between goals, tasks, and bond. These factors, however, were very highly correlated (0.708–0.964) as shown previously [101, 104, 105], raising questions about their interpretation, and limiting their use as simultaneous predictors in regression models because of multicollinearity concerns. Literature on the factor structure of the WAI(-S) in contexts other than coaching has yielded similarly inconsistent results, suggesting either a one- [104, 106], two- [63, 107], or three-factor structure [63, 108]. Indeed, a more recent study [109] has suggested co-existence of general and specific working alliance factors but advised caution in the interpretation of the specific factors because they lacked uniqueness. Based on our own data, we are inclined to follow this suggestion. Thus, while the working alliance is theoretically assumed to consist of three distinct factors, the items of the WAI(-S) may lack sufficient factor-specific heterogeneity to accurately distinguish between these factors, at least in the context of coach-coachee relationships [109, 110]. Alternatively, and/or additionally, the distinction between goals, tasks, and bond may be less pronounced in coaching practice than theory assumes. Taken together, across various types of coaching, the quality of the overall working alliance seems to be a significant, modest to moderate predictor of goal attainment, but it is less clear which (unique) role different aspects of the working alliance play in explaining this effect.

#### Basic psychological need satisfaction and goal attainment

Like the prospective effect of working alliance quality, basic psychological need satisfaction at baseline explained a rather modest but significant 2.9% of variance in goal attainment at follow-up, on top of the variance explained by a priori covariates. Again, considering our inconsistent factor analytical results, and very high correlations between different need satisfaction factors (0.774–0.932), it remains unclear whether basic psychological need satisfaction in the coaching context, measured with BPNs-COACH, is indeed best represented multidimensionally. Theoretically, the three factors are however considered distinct and non-compensatory yet interdependent [47, 54], and exploratory analyses showed that satisfaction of all three needs—autonomy, competence, relatedness—was significantly associated with goal attainment in the current sample.

Previous research has suggested that fulfilment of the need for autonomy is essential in the context of goal attainment [44, 49, 53, 111]. Actions generated by own motives, interests, and values (i.e., the self-concordance of goals [112]) enhance intrinsic motivation to goal attainment [21, 43, 53], which increases the likelihood of sustained effort, and use of the acquired knowledge outside the coaching context [40]. Fulfilment of the need for competence, the propensity to be interested and determined in learning new skills, has also been identified as an important factor in understanding the dynamics of motivation to attain goals [44, 113, 114]. Competence includes appraisal of the personal importance of the task and is in this sense distinct from self-efficacy [115]. Though both constructs are often used interchangeably, self-efficacy is the experienced confidence to persevere in completing a task without considering expected outcomes. In our study, competence satisfaction was measured by four items: “I became confident that I could successfully complete difficult tasks”, “I felt like I could do only a few things right” (reverse coded), “I was strengthened in the belief in my own abilities”, and “I felt I was competent to achieve my goals”. While the last two items more closely capture core aspects of the need for competence, since they included the (individual) goals pursued, the first two items may have tapped more into aspects of global self-efficacy.

Satisfaction of the need for autonomy and competence are both required to maintain behaviour that is needed to attain goals, though both needs may differ in their effect on motivation. Perceived competence is essential for any kind of motivation, while perceived autonomy is indispensable for motivation to be intrinsic [44]. In line with this assumption, autonomy satisfaction has been shown to be positively associated with autonomous motivation and negatively with controlled motivation, whereas competence satisfaction relates positively to both types of motivation [116]. The type of support is important too, and coaching (i.e., a non-directive approach to achieve goals) and training (i.e., guiding with advice to improve skills) address different needs [100]. The higher the need for autonomy at the start of a trajectory, the more this need has been shown to be fulfilled by coaching, resulting in higher rates of goal attainment and coaching satisfaction. The higher the need for competence, the more this need was fulfilled by training, leading to higher levels of training satisfaction and intrinsic motivation to perform. Thus, the need for autonomy is possibly met to a greater extent through coaching, and the need for competence through training.

Lastly, relatedness satisfaction, the fundamental need to feel connected to others, was positively associated with goal attainment in our data. According to relationships motivation theory [55], one of the self-determination mini theories, it is mainly this need that motivates people to engage in interactions and relationships with others. However, high quality relationships do not only meet the need for relatedness, but also satisfy the need for autonomy and competence [55], in line with our observation that all three basic needs are prospectively associated with goal attainment. More specifically, recent work [117] has suggested that relatedness and autonomy satisfaction interactively explain relationship quality, such that relatedness satisfaction is positively associated with relationship quality, but especially when autonomy satisfaction is also high. Translated to the context of coaching, interactions between coach and coachee may be particularly motivating and fruitful when the coachee feels related to the coach and at the same time autonomous.

#### Other outcome indicators

The working alliance quality and basic psychological need satisfaction at baseline were not associated with wellbeing, (absence of) psychopathology, and personal growth initiative. This was not in line with our expectations, considering previous work showing that working alliance quality is not merely associated with results outcomes in coaching, but also with affective and cognitive outcomes [26, 118, 119]. Likewise, basic psychological need satisfaction [47, 120–122] has been associated with various aspects of wellbeing and illbeing, and we expected to find similar associations in our data. However, it is important to consider that coaching trajectories are highly individualized in terms of their desired outcomes, since these are dependent on the specific goals set by the coachee [29, 123, 124]. Because of the primary focus on facilitating the coachee to formulate and reach these goals, the effectiveness of coaching trajectories relates most directly to goal attainment as outcome [125], and less directly to more global outcome domains, such as wellbeing, (absence of) psychopathology, and personal growth initiative. Consequently, what happens between the coach and the coachee may be more directly reflected in goal attainment results than in these other outcome domains.

In addition, the three-week interval may have made it difficult to observe significant changes in wellbeing, (absence of) psychopathology, and personal growth initiative. Previous studies have suggested that the more sessions take place between two measurements, the more coachees are actively involved in change and development [31, 126]. Since our study used a relatively short measurement interval, relevant changes in some outcome domains may not have been captured, which may also have impacted results. Moreover, instead of applying simple correlation analyses, we used multiple regression techniques to look specifically at the amount of variance in outcome indicators explained uniquely by working alliance and basic psychological need satisfaction, on top of the variance already explained by levels of the dependent variable at baseline, demographic factors, and number of coaching sessions. Thus, we used a more specific and rigorous analyses strategy that considered autocorrelation in the outcome measures, which may also explain discrepancies with previous correlational study findings (e.g., [26]).

### Implications

Coachees who indicated a higher degree of satisfaction of the need for relatedness at baseline, provided higher ratings of the quality of working alliance at follow-up. Moreover, the perception of the affective bond with the coach best predicted goal attainment. Hence, establishing a good coach-coachee relationship is the first and perhaps most important task in the coaching process [27, 127]. To build a sturdy basis, the coach’s focus on relational behaviours is essential; unconditional acceptance and support of the coachee, empathy and congruence [128]. These attitudes may be evaluated by collecting and delivering feedback to the coachee [129, 130], and further developed through inter- and supervision. Moreover, previous research [129] recommends to adapt or tailor sessions to coachees’ specific characteristics. The same applies to need fulfilment; individuals differ in the degree they seek and demand need fulfilment [114], and needs may differ qualitatively between individuals and contexts, which is an important notion for coaches as this also requires a highly personalised approach of need support. Different techniques may promote motivation and behavioural outcomes by tailoring support to the coachee’s personal needs [131].

Techniques to support autonomy include facilitating ownership and personal endorsement of a task or behaviour. To support competence, techniques include communicating clear expectations, guiding skill development, and providing constructive feedback. Regarding relatedness support, it is important to foster a sense of interpersonal connectedness, for instance through enhancing the coachee’s feeling of being accepted and understood.

More research is needed to unravel the complex interactions of working alliance and basic psychological need satisfaction in coaching [132], and how these factors are prospectively associated with coaching outcomes. In particular, prospective and experimental investigations of the role of basic psychological need satisfaction as common factor in coaching are much needed [18, 51, 53]. As pointed out previously (e.g., [133]), standard factor analytical procedures, such as those applied in the current study, may not suffice to adequately model the underlying structure of measures such as the WAI(-S), as they are not able to take into account the co-existence of global and specific factors. More recently developed methods, such as bifactor exploratory structural equation modelling [133], may help to elucidate the underlying structure of these measures further. However, the current findings also shed a new light on existing definitions and conceptualizations of the working alliance in coaching, and more research is needed to further integrate different perspectives, and better measure and understand the unique contributions of specific relational and motivational factors to outcomes in coaching.

### Strengths and limitations

The current study adds to the literature on the interplay of working alliance and basic psychological need satisfaction in the context of coaching. The main strength is its longitudinal design, with baseline and follow-up measurements, thus allowing to examine associations between constructs over time. Further strengths lie in the inclusion of a broad range of coaching contexts and settings, and the multidimensional outcome assessment (i.e., goal attainment, wellbeing, (absence of) psychopathology, and personal growth initiative). Nonetheless, several limitations should be kept in mind when interpreting the findings and, moreover, are important to address in future studies.

Firstly, women were overrepresented in our study sample, and most subjects were highly educated. Thus, our sample is not representative of the general population. However, a large global study [134] among 2,165 coachees from 64 countries showed a similar overrepresentation of females and highly educated individuals. In addition, among various types of coaching, life coaching was most common among respondents from this study, very much in line with what was observed in our dataset. Thus, while not accurately reflecting demographic characteristics at the general population level, our sample does appear representative of the global coachee population.

Second, it should be noted that our study did not utilize a strict pre-post design, and the number of sessions between measurements varied between respondents, which may have affected results and hampers comparability with other studies (see also: [135]). In addition, assessments were three weeks apart, and it remains unclear to what extent our findings would reproduce beyond this specific time interval.

Third, goal attainment or progress was assessed subjectively, and we cannot exclude the possibility of our findings being biased by some form of social desirability in the self-reports. Although participation in the study was on a voluntary basis, and data collection was online and anonymous, coachees may have overreported socially desirable attitudes or behaviours, for instance due to feelings of loyalty or sympathy towards the coach. It should also be noted that all study variables were constructed from coachee ratings, based on previous findings suggesting that the client or coachee perspective is most crucially related to successful outcomes [17, 36, 37, 56]. However, the coaching relationship is based on mutual trust and agreement between coach and coachee, and future studies should consider obtaining additional information from sources other than the coachee in order to provide a more complete picture of relational and motivational processes in coaching.

Fourth, we conceptualized the working alliance and basic psychological need satisfaction as unidimensional constructs for our main analyses, and only looked exploratively at their separate factors because these were very highly correlated. Thus, interpretations regarding the unique contributions of these separate factors remain speculative based on our data.

Fifth, our study employed a two-wave observational design, which did not allow for testing of relevant mediational models, since there is consensus that this requires at least three separate measurements, and preferably experimental manipulation(s) [136, 137]. In addition, while our findings provide insight in associations between variables over time, an experimental setup would be necessary to meet all criteria for testing causality.

Lastly, although all coaching outcome indicators—i.e., goal attainment, wellbeing, (absence of) psychopathology, and personal growth initiative—significantly improved over time, it is important to underline that these changes cannot be attributed to coaching per se, due to lack of a control condition. Indeed, the purpose of this study was to evaluate factors associated with coaching outcomes within a heterogeneous group of coachees, rather than to isolate effects that are specific to coaching interventions.

## Conclusion

This study used the self-determination theory, a macro theory of human motivation, development, and health [23], to unravel aspects of the coach-coachee relationship and their association with coaching outcome indicators. The results suggest that a sense of basic psychological need fulfilment in the coaching context may facilitate the establishment of a good working alliance. In addition, our findings support the idea that coachees who regard the relationship with their coach as more engaging and psychologically fulfilling, are more likely to feel that they reached their goal*s* through coaching. However, contrary to expectations, these relational aspects were not predictive of wellbeing, (absence of) psychopathology, or personal growth initiative, thus challenging the view that such coaching outcomes are critically driven by the coaching relationship per se. Profound knowledge of the mechanisms involved is essential to better understand processes of change and development to increase effectiveness of coaching, and to refine theories of human motivation in attaining goals. More detailed, multi-rater assessments, in addition to better controlled experimental and multi-wave study designs, may help to further substantiate the current findings, and unravel the complex interplay of relational and motivational factors in coaching.

## Supplementary Information

**Additional file 1. BPNs-COACH in Dutch.** Dutch version of the Basic Psychological Needs in Coaching relationships scale (BPNs-COACH) to assess coachee basic psychological need satisfaction in coaching relationships.

**Additional file 2. BPNs-COACH in English.** English translation of the Dutch version of the Basic Psychological Needs in Coaching relationships scale (BPNs-COACH) to assess coachee basic psychological need satisfaction in coaching relationships.

## Data Availability

The dataset is available from the corresponding author on reasonable request.

## Abbreviations

SDT: Self-determination theory
WAI-S: Short Working Alliance Inventory
BPNs-COACH: Basic Psychological Needs in Coaching relationships questionnaire
BPNS: Basic Psychological Needs Scale
BPNSFS: Basic Psychological Need Satisfaction and Frustration Scale
HCCQ: Health Care Climate Questionnaire
MHC-SF: Mental Health Continuum Short Form
MHI-5: Mental Health Inventory
PGIS: Personal Growth Initiative Scale
KMO: Kaiser–Meyer–Olkin measure
CFA: Confirmatory factor analysis
RMSEA: Root mean squared error of approximation
CFI: Comparative fit index
SRMR: Standardized root mean squared residual
pFDR: False discovery rate corrected p-value
pFWE: Familywise error-corrected p-value
EFA: Exploratory factor analysis
