# Supplementary material for: Prospective associations between working alliance, basic psychological need satisfaction, and coaching outcome indicators: a two-wave survey study among 181 Dutch coaching clients
Source: BMC Psychol. 2022 Nov 15;10:269. doi: 10.1186/s40359-022-00980-9 (PMC9664732; doi:10.1186/s40359-022-00980-9)
Supplement: Supplementary file 1 — Additional file 1. BPNs-COACH in Dutch. Dutch version of the Basic Psychological Needs in Coaching relationships scale (BPNs-COACH) to assess coachee basic psychological need satisfaction in coaching relationships. [file 40359_2022_980_MOESM1_ESM.pdf]

Additional file 1

BPNs-COACH in Dutch

Dutch version of the Basic Psychological Needs in Coaching relationships scale (BPNs-COACH) to assess coachee basic psychological need satisfaction in coaching relationships.

Introductie

Lees onderstaande stellingen zorgvuldig door.

Denk daarbij (terug) aan uw coachtraject.

		nooit	zelden	soms	regel-<br>matig	vaak	altijd
1	Ik voelde me begrepen	0	1	2	3	4	5
2	Ik voelde me onzeker over mijn vaardigheden	0	1	2	3	4	5
3	Ik had een gevoel van keuze en vrijheid in de dingen die ik ondernam	0	1	2	3	4	5
4	Ik had het gevoel slechts oppervlakkig contact te hebben (met mijn coach)	0	1	2	3	4	5
5	Ik kon niet zeggen wat ik echt wilde	0	1	2	3	4	5
6	Ik had het gevoel dat er om mij werd gegeven	0	1	2	3	4	5
7	Ik kreeg het vertrouwen dat ik ook moeilijke taken met succes kon voltooien	0	1	2	3	4	5
8	De besluiten die ik nam, weerspiegelden echt wat ik wil(de)	0	1	2	3	4	5
9	Ik kreeg het gevoel dat ik maar weinig dingen goed kon doen	0	1	2	3	4	5
10	Ik werd afstandelijk bejegend	0	1	2	3	4	5

11	Ik werd gestimuleerd om mijn eigen keuzes te maken	0	1	2	3	4	5
12	Ik voelde me verplicht om gegeven adviezen op te volgen	0	1	2	3	4	5
13	Ik werd geaccepteerd zoals ik was/ben	0	1	2	3	4	5
14	Het geloof in mijn eigen kunnen werd versterkt	0	1	2	3	4	5
15	Ik kreeg het gevoel in staat te zijn om mijn doelen te bereiken	0	1	2	3	4	5

Scoring Autonomy satisfaction: items 3, 5*,8, 11, 12*

Scoring Competence satisfaction: items 2*[,a], 7, 9*, 14, 15

Scoring Relatedness satisfaction: items 1, 4*, 6, 10*, 13

* reverse coded

[a] item omitted due to low factor loadings (< .40)
