# Supplementary material for: Prospective associations between working alliance, basic psychological need satisfaction, and coaching outcome indicators: a two-wave survey study among 181 Dutch coaching clients
Source: BMC Psychol. 2022 Nov 15;10:269. doi: 10.1186/s40359-022-00980-9 (PMC9664732; doi:10.1186/s40359-022-00980-9)
Supplement: Supplementary file 2 — Additional file 2. BPNs-COACH in English. English translation of the Dutch version of the Basic Psychological Needs in Coaching relationships scale (BPNs-COACH) to assess coachee basic psychological need satisfaction in coaching relationships. [file 40359_2022_980_MOESM2_ESM.pdf]

Additional file 2

BPNs-COACH in English

English translation of the Dutch version of the Basic Psychological Needs in Coaching relationships scale (BPNs-COACH) to assess coachee basic psychological need satisfaction in coaching relationships.

Introduction

Please read each of the following items carefully.

Think about your coaching trajectory.

		never	rarely	some-times	regu-larly	often	always
1	I felt understood	0	1	2	3	4	5
2	I felt insecure about my abilities	0	1	2	3	4	5
3	I felt a sense of choice and freedom in the things I undertook	0	1	2	3	4	5
4	I had the feeling that I had only superficial contact (with my coach)	0	1	2	3	4	5
5	I couldn’t say what I really wanted	0	1	2	3	4	5
6	I felt I was cared for	0	1	2	3	4	5
7	I became confident that I could successfully complete difficult tasks	0	1	2	3	4	5
8	The decisions I made reflected what I really want(ed)	0	1	2	3	4	5
9	I felt like I could do only a few things right	0	1	2	3	4	5
10	I was treated cold and distant	0	1	2	3	4	5
11	I was encouraged to make my own decisions	0	1	2	3	4	5
12	I felt obliged to follow given advice	0	1	2	3	4	5

13	I was accepted for who I was/am	0	1	2	3	4	5
14	I was strengthened in the belief in my own abilities	0	1	2	3	4	5
15	I felt I was competent to achieve my goals	0	1	2	3	4	5

Scoring Autonomy satisfaction: items 3, 5*,8, 11, 12*

Scoring Competence satisfaction: items 2*,[a], 7, 9*, 14, 15

Scoring Relatedness satisfaction: items 1, 4*, 6, 10*, 13

* reverse coded

[a] item omitted due to low factor loadings (< .40)
